# 2011年美国临床肿瘤学会年会——小细胞肺癌治疗研究进展

**DOI:** 10.3779/j.issn.1009-3419.2011.11.07

**Published:** 2011-11-20

**Authors:** 红阳 卢, 胜林 马

**Affiliations:** 1 310022 杭州，浙江省肿瘤医院，浙江省胸部肿瘤诊治技术研究重点实验室 Zhejiang Key Labriary of Diagnisis and Treatment Technology on Thoracic Oncology, Zhejiang Cancer Hospital, Hangzhou 310022, China; 2 310022 杭州，浙江省肿瘤医院，肿瘤内科 Department of Medical Oncology, Zhejiang Cancer Hospital, Hangzhou 310022, China; 3 310006 杭州，杭州市第一人民医院 Department of Medical Oncology, Hangzhou First People's Hospital, Hangzhou 310006, China

第47届美国临床肿瘤学会（American Society of Clinical Oncology, ASCO）年会于2011年6月3日-7日在美国芝加哥召开。现就本次大会关于小细胞肺癌（small cell lung cancer, SCLC）治疗的研究进展作一介绍。

## 1 一线治疗

同步放化疗是局限期SCLC的标准治疗，但仍然会因原发灶或纵隔淋巴结复发而导致治疗失败，外科手术是肺癌最有效的局部治疗方法，手术治疗在局限期SCLC治疗中的作用值得研究。Tashi等^[1]^回顾性分析了手术切除在局限期SCLC治疗中的作用，共有8, 791例患者，手术切除915例（10.4%），化疗或放疗及放化疗者5, 865例（66.6%），未接受任何治疗2, 011例（22.9%）。915例手术治疗的患者中，Ⅰ期占65.8%，Ⅱ期占13.4%，Ⅲ期占20.8%;肺叶切除加淋巴结清扫占46.3%，局部切除占21.1%，单纯肺叶切除占20.5%，全肺切除占6.9%。研究结果显示：与非手术切除患者相比，手术切除患者的生存期明显提高，手术患者的中位生存期（median survival time, MST）为38.7个月，而非手术患者为12.4个月（*P*=0.000, 1），Ⅰ期患者为45.9个月*vs* 15.9个月，Ⅱ期为39.4个月*vs* 13.7个月，Ⅲ期为21.8个月*vs* 11.5个月（*P*=0.000, 1）; 手术后行放化疗的患者生存期明显好于单纯放化疗患者（30.9个月*vs* 14.7个月，*P*=0.000, 1）。该作者认为，局限期SCLC患者接受手术治疗能提高生存期，手术治疗应成为早期患者的选择。该临床研究认为Ⅲ期SCLC亦有手术价值，这可能与手术组和非手术组患者间的一般情况、辅助治疗手段可能存在差异有关，且该研究仅是回顾性研究，仍需前瞻性研究来证实。Varlotto等^[2]^对2, 214例Ⅰ期和Ⅱ期SCLC进行回顾性分析，结果显示肺叶切除或更大的切除后未行辅助放疗的患者在MST方面要优于亚叶切除后未行辅助放疗的患者及单纯放疗的患者（50个月*vs* 30个月，*P*=0.006;50个月*vs* 20个月，*P* < 0.000, 1）; 未进行术后辅助放疗的亚叶切除患者MST优于单纯放疗的患者（*P*=0.002）。目前对于肿瘤大小为T1或T2且无淋巴结转移患者，美国国家综合癌症网（National Comprehensive Cancer Network, NCCN）SCLC指南（2011版）认为可以考虑先行肺叶切除加纵隔淋巴结清扫术，术后淋巴结阴性者行化疗，淋巴结阳性者行同步的纵隔放疗和化疗。

局限期SCLC患者主张进行早期同步放化疗，但放化疗后的毒性一直是人们关注的焦点。Salama等^[3]^对局限期SCLC同步放化疗的3项临床研究的数据进行了合并分析，共有211例患者接受了治疗，所有患者在2个周期的化疗后接受同步放化疗，放疗剂量为70 Gy，100例患者进行了放疗剂量-容积参数及不良事件的评估。研究结果显示，肺毒性的发生较少见，研究中只有3例患者出现肺毒性，老年患者（*P*=0.09）、肺总容量较小（*P*=0.05）、肺暴露低剂量（平均V5=70%，*P*=0.09;平均V10=63%，*P*=0.07）、中剂量（平均V20=50%，*P*=0.04）和高剂量（平均V60=25%，*P*=0.01）与肺毒性的发生有相关性，但该研究统计分析的力度不足。目前广泛接受的局限期SCLC同步放化疗指在诱导化疗开始后的30天内开始放疗，或化疗开始后的2个周期内进行放疗，且放疗越早介入，疗效越好。而Salama等^[3]^研究治疗方案为化疗2个周期后开始放疗，这样的研究得出肺毒性相关性的数据的参考价值有限。

顺铂与卡铂被广泛用于SCLC的一线化疗，Rossi等^[4]^进行了含顺铂或卡铂方案一线治疗SCLC的*meta*分析，共有来自4项临床研究的663例患者（329例患者接受含顺铂方案，334例为含卡铂方案），两组的基线特征是均衡的。结果显示两组的MST均为9.5个月，且根据性别、年龄、分期及体能状况（performence status, PS）评分均无差异; 两组的有效率分别为67.5%和65.6%（*P*=0.66）; 中位无进展生存期（progression-free survival, PFS）为5.3个月和5.5个月（*P*=0.90）; 血液学毒性卡铂组高，非血液学毒性顺铂组高。老年患者年龄大，合并症多，接受化疗是否能延长生存期受到人们的日益关注，Caprario等^[5]^进行了老年患者是否需接受化疗及其作用的回顾性分析研究，从1992年-2001年共10, 428例年龄 > 65岁的患者入组，接受化疗者占67.1%，放疗者占39.1%，手术者占3.4%，未治疗者占21.8%，主要的化疗方案为足叶乙甙联合顺铂或卡铂，年龄 > 85岁者相比于65岁-69岁者较少接受化疗。研究结果显示：MST为7个月; 与提高生存期相关的因素有女性、黑色人种、局限期、接受过治疗及较少合并症; 即使经过分期调整后接受化疗者，MST仍能提高6个月。笔者认为对于老年患者也应考虑化疗，但需关注化疗的耐受性及可能出现的不良反应。

Obatoclax（Ob）是BCL-2拮抗剂，Langer等^[6]^进行了Ob联合CE方案（足叶乙甙联合卡铂）治疗初治广泛期SCLC的随机对照Ⅱ期临床研究。CE组为足叶乙甙100 mg/m^2^/d，连用3天，卡铂按药物浓度-时间曲线下面积（area under concentration-time curve, AUC）=5计算，CEOb组在CE方案基础上加Ob 30 mg/d，连用3天，化疗6个周期后Ob维持治疗。共165例患者入组，两组在性别、PS评分及转移情况方面均衡。研究结果显示：CEOb组有效率为64.9%，CE组为53.8%（*P*=0.11）; CEOb组PFS为6.0个月，CE组为5.4个月（*P*=0.08）; CEOb组1年生存率为45.5%，CE组为37.2%（*P*=0.19）; CEOb组MST为10.6个月，CE组为9.9个月（*P*=0.050, 6）; PS评分为0分或1分的患者中，CEOb组MST为11.9个月，CE组为10.1个月（*P*=0.050, 2）; 两组3级-4级的血液学毒性相似。CEOb组有延长生存期的趋势，值得进一步开展Ⅲ期随机对照研究。

## 2 二线治疗

近年来氨柔比星在SCLC中的应用受到日益关注，托泊替康是美国食品药品监督管理局唯一批准用于SCLC二线治疗的药物。Jotte等^[7]^进行了氨柔比星或托泊替康二线治疗SCLC的Ⅲ期随机对照研究，637例患者按2:1进入氨柔比星组（424例）与托泊替康组（213例），氨柔比星的用法为40 mg/m^2^/d，连用3天，托泊替康1.5 mg/m^2^/d，连用5天，终点观察指标为生存期、缓解率、PFS及安全性，两组的基线特征相似。结果显示：氨柔比星组的MST为7.5个月，托泊替康组为7.8个月（*P*=0.17）; 氨柔比星组的客观缓解率为31.1%，托泊替康组为16.9%（*P*=0.000, 1）; 氨柔比星组的缓解持续时间为4.8个月，托泊替康组为4.2个月（*P*=0.009）; 氨柔比星组的PFS为4.1个月，托泊替康组为3.5个月（*P*=0.018）; 在敏感复发患者中氨柔比星组的MST为9.2个月，托泊替康组为9.9个月（*P*=0.616）; 在难治性患者中氨柔比星组的MST为6.2个月，托泊替康组为5.7个月（*P*=0.047）; 3级-4级级的不良事件为中性粒细胞减少为41% *vs* 53%，血小板减少为21% *vs* 54%，贫血为16% *vs* 30%，感染为16% *vs* 10%及中性粒细胞减少性发热为10% *vs* 4%（*P*值均为0.05）; 心脏事件均为5%;输血率为32% *vs* 53%（*P*=0.01）。氨柔比星比托泊替康有效率高，在难治性患者中氨柔比星组的MST更长。笔者认为在应用氨柔比星时需密切注意其血液学毒性，并积极对症治疗。口服托泊替康有严重的血液学毒性，常规用法为2.3 mg/m^2^/d，连用5天。Kontopodis等^[8]^进行了口服托泊替康每周给药治疗复发SCLC的Ⅰ期临床研究，以明确剂量限制性毒性（dose-limiting toxicities, DLTs）及最大耐受剂量（maximum tolerated dose, MTD），共有18例患者入组，托泊替康第1、8、15天给药，28天重复，从每天3 mg/m^2^开始，每次增加0.5 mg/m^2^，直至MTD。研究结果显示：MTD为4 mg/m^2^，Ⅱ期临床研究的推荐剂量为4 mg/m^2^，第1、8、15天使用，28天重复。改变托泊替康给药方式是否能降低其毒性及对生存期的影响等有待研究。NK012是拓扑异构酶Ⅰ抑制剂，能使7-乙基-10-羟基喜树碱（7-ethyl-10-hydroxycamptothecin, SN-38）持续而缓慢地释放。伊立替康（irinotecan, CPT-11）是无活性的前药，需经羧酸酯酶的活化转变为其活性代谢产物SN-38而发挥效用。尿苷二磷酸葡糖醛酰转移酶（UGTs）分为UGT1和UGT2两个家族，UGT1A1是*UGT1*基因的一个亚型，SN-38通过肝脏UGT1A1的糖基化作用转变为无活性的SN-38G。*UGT1A1*基因的变异型UGT1A1^*^28与UGT1A1表达下降有关，可导致活性代谢产物SN-38的明显增加。Raefsky等^[9]^进行了NK012治疗含铂方案化疗后复发SCLC的Ⅱ期临床研究，入组需排除有脑转移及曾用拓扑异构酶Ⅰ治疗的患者，每28天给药1次。根据*UGT1A1*基因型分别给予28 mg/m^2^（野生型）或18 mg/m^2^（UGT1A1^*^28型）。结果显示：40例敏感复发患者入组已超过11个月，难治性组仍在入组中; 敏感复发患者的有效率为22%（包括2例完全缓解），疾病控制率为68%;3级-4级的毒性包括血小板减少（13%），中性粒细胞减少（44%），贫血（5%）及腹泻（8%），没有治疗相关性死亡。由于NK012对敏感复发SCLC有较好的疗效及耐受性，与顺铂联合应用的研究正在进行中。

NGR-hTNF是一种选择性血管靶向药物，能提高肿瘤组织内阿霉素的渗透性并降低肿瘤组织内压力。Vigano等^[10]^进行了NGR-hTNF联合阿霉素治疗复发SCLC的Ⅱ期临床研究，用法为0.8 g/m^2^，直至疾病进展，阿霉素75 mg/m^2^，每3周重复，上限总量为550 mg/m^2^。共28例患者入组，16例患者为顺铂耐药，12例患者对顺铂敏感。研究结果显示：NGR-hTNF并未增加阿霉素相关的毒性，3级-4级NGR-hTNF相关毒性未出现，1级-2级的相关毒性主要为短暂性发热（61%）; 疾病控制率为55%，包括6例部分缓解及9例疾病稳定; 中位PFS为3.2个月; 随访19.3个月后，6个月及1年的生存率分别为49%和34%;亚组分析显示：顺铂耐药及顺铂敏感患者的有效率分别为19%和27%，中位PFS分别为2.7个月和4.1个月，1年生存率分别为27%和42%。NGR-hTNF联合阿霉素治疗复发SCLC值得进一步研究。索坦（sunitinib）是一多靶点酪氨酸激酶抑制剂，有抗血管生成及直接抗肿瘤作用。Han等^[11]^进行了索坦治疗复发或难治性SCLC的Ⅱ期临床研究，主要评价疗效及安全性。索坦用法为50 mg/d，连用4周，停2周，6周为1个周期，共25例患者入组，24例患者接受治疗，2例敏感复发患者疗效达部分缓解，7例疾病稳定，中位PFS和MST分别为1.4个月和5.6个月。3/4级的毒性包括血小板减少（63%），中性粒细胞减少（25%），无力（8%）及厌食（8%），46%的患者剂量下调1次或2次。由此可见，索坦治疗复发SCLC有一定疗效，但血小板下降发生率较高。

## 3 预防性全脑照射

Schild等^[12]^对化疗或放化疗后疗效达疾病稳定及以上患者进行了预防性全脑照射（prophylactic cranial irradiation, PCI）的汇总分析，共有来自4项Ⅱ期临床研究的318例广泛期患者及421例局限期患者，其中459例患者接受PCI（30 Gy/15F或25 Gy/10F），280例未接受PCI。研究结果显示，PCI明显提高了生存期（*P*=0.000, 1），PCI组1年和3年的生存率分别为73%和20%，未接受PCI组则为52%和6%;PCI组3级不良事件发生率为64%，未接受PCI组为50%（*P*=0.009, 6），PCI组的主要不良事件为脱发、贫血、食管炎及昏睡，食管炎同治疗顺序相关，其见于胸部放疗同步PCI的患者; 局限期患者中PCI剂量为25 Gy/10F的生存期比接受30 Gy/15F的患者（*P*=0.051, 2）长，在广泛期SCLC中并没有显示出25 Gy/10F的优势。该作者认为，PCI对于化疗或放化疗达疾病稳定及以上SCLC患者能提高生存期，剂量分割方案在其中显示出了重要性。Slotman等^[13]^报道了一项接受4个-6个周期化疗且有效的广泛期SCLC预防性全脑照射的Ⅲ期随机对照研究，286例患者入组。结果显示：1年脑转移的发生率PCI组为14.6%，对照组为40.4%;MST PCI组为6.7个月，对照组为5.4个月; 1年生存率PCI组为27.1%，对照组为13.3%。Le Péchoux等^[14]^进行了对化疗和放疗后达完全缓解的局限期SCLC患者行高剂量（36 Gy）PCI或标准剂量（25 Gy）PCI的随机对照研究，结果显示高剂量并不能降低脑转移的发生率，但增加了死亡率。对治疗有效的局限期或广泛期SCLC患者美国NCCN的指南（2011版）推荐行PCI，剂量为25 Gy/10F或30 Gy/15F。Schild等^[12]^研究未对有效患者及疾病稳定患者进行分层，且该研究为汇总分析，而非Ⅲ期随机对照研究，因此对疗效达疾病稳定的患者是否需要接受PCI治疗有待进一步研究。

## 4 基础研究

整合素-1与SCLC的高度侵袭性及转移有关，E-钙粘蛋白能减少转移，Rac1蛋白与细胞的粘附、迁移、浸润及转移有关。Chang等^[15]^进行了整合素-1、E-钙粘蛋白及Rac1蛋白与SCLC预后的相关性研究。免疫组化检测了112例患者的上述3个指标，广泛期65例，局限期47例，中位随访61个月。研究结果显示：整合素-1、E-钙粘蛋白及Rac1蛋白表达的SCLC分别为64例、73例和99例; E-钙粘蛋白的表达与生存期相关; 整合素-1及Rac1蛋白的表达与生存期或PFS无关。亚组分析显示，在 < 2个转移灶的患者中，整合素-1表达的患者生存期长于无表达患者（*P*=0.047）; 在男性和不吸烟患者中E-钙粘蛋白表达患者的生存期长于不表达患者。多因素分析显示：局限期、总有效率及E-钙粘蛋白是有较好生存期的独立预后因子，局限期、总有效率及 < 2个转移灶是具有较长PFS的独立预后因子。Chang认为，E-钙粘蛋白可能是SCLC的预后因子。

Wang等^[16]^进行了腺病毒介导siRNA靶向*c-Met*基因抑制SCLC细胞增殖与侵袭的研究。研究结果显示，SCLC肿瘤组织c-Met mRNA及蛋白的表达明显高于相应的肺部非肿瘤组织（*P* < 0.05），腺病毒介导siRNA靶向*c-Met*基因在体内与体外均能下调SCLC c-Met蛋白的表达，进而影响侵袭和转移。笔者认为，抑制*c-Met*基因或许是SCLC治疗的一个重要方向。

## 5 结语

综上所述，肿瘤大小为T1或T2且无淋巴结转移的局限期SCLC患者可以考虑先行肺叶切除加纵隔淋巴结清扫术，再行化疗或放化疗，手术在局限期SCLC治疗中的地位值得进一步评价。经过化疗或放化疗后疗效达疾病稳定的患者是否需要行PCI治疗值得进一步研究，有待更多数据证实。顺铂与卡铂作为SCLC的一线化疗药物疗效相当，但毒性有差异。老年SCLC患者也应考虑化疗，但需关注化疗的耐受性及可能出现的不良反应。氨柔比星在SCLC的二线治疗中有较好疗效，但需注意其血液毒性。靶向药物NGR-hTNF及obatoclax对化疗有增效作用，有应用前景。E-钙粘蛋白可能是SCLC的预后因子，抑制*c-Met*基因可能会成为SCLC治疗的一个方向。
